# A DKP Cyclo(L-Phe-L-Phe) Found in Chicken Essence Is a Dual Inhibitor of the Serotonin Transporter and Acetylcholinesterase

**DOI:** 10.1371/journal.pone.0050824

**Published:** 2012-11-28

**Authors:** Nobuo Tsuruoka, Yoshinori Beppu, Hirofumi Koda, Nobutaka Doe, Hiroshi Watanabe, Keiichi Abe

**Affiliations:** 1 BRAND’S Brain Research Centre, Cerebos Pacific Limited, Singapore, Singapore; 2 Frontier Center for Value Creation, Suntory Business Expert Limited, Osaka, Japan; 3 Section of Behavioral Science, Kouiken Co. Ltd., Hyogo, Japan; 4 Laboratory of Neurogenesis and CNS Repair, Institute for Advanced Medical Science, Hyogo College of Medicine, Hyogo, Japan; 5 Institute for Health Care Science, Suntory Wellness Limited, Tokyo, Japan; University of Pittsburgh, United States of America

## Abstract

Diketopiperazines (DKPs) are naturally-occurring cyclic dipeptides with a small structure and are found in many organisms and in large amounts in some foods and beverages. We found that a chicken essence beverage, which is popular among Southeast Asians as a traditional remedy and a rich source of DKPs, inhibited the serotonin transporter (SERT) and suppressed serotonin uptake from rat brain synaptosomes, which prompted us to isolate and identify the active substance(s). We purified a SERT inhibitor from the chicken essence beverage and identified it as the DKP cyclo(L-Phe-L-Phe). Interestingly, it was a naturally occurring dual inhibitor that inhibited both SERT and acetylcholinesterase (AChE) *in vitro*. The DKP increased extracellular levels of the cerebral monoamines serotonin, norepinephrine, and dopamine in the medial prefrontal cortex and acetylcholine in the ventral hippocampus of freely moving rats when administered orally. Moreover, cyclo(L-Phe-L-Phe) significantly shortened escape latency in the water maze test in depressed mice previously subjected to a repeated open-space swimming task, which induces a depression-like state. Cyclo(L-Phe-L-Phe) also significantly improved accuracy rates in a radial maze test in rats and increased step-through latencies in a passive avoidance test in mice with scopolamine-induced amnesia. These animal test results suggest that cyclo(L-Phe-L-Phe), which is present abundantly in some foods such as chicken essence, may abrogate the onset of depression and, thus, contribute to preventing the development of Alzheimer’s disease and other dementia, because senile depression is a risk factor for dementia.

## Introduction

Cyclic dipeptides, or 2,5-diketopiperazines (DKPs), are found endogenously in many organisms and in large amounts in some foods and beverages [1], [2], *e.g.*, aged sake, beer, cocoa, roasted coffee, roasted malt, dried squid, and chicken essence [3]–[11]. DKPs are easily formed through nonenzymatic dehydration and condensation of two N-terminal amino acid residues of linear peptides or proteins during storage (*e.g.*, fermented foods) and through food sterilization (*e.g.*, cooked foods). Proline-based DKPs such as cyclo(L-Pro-L-Leu), cyclo(L-Pro-L-Phe), cyclo(L-Pro-L-Pro), and cyclo(L-Pro-L-Val) are bitter components of beer, coffee, and cocoa [3]–[9]. DKPs and their derivatives exhibit various biological activities, including antiviral, antibacterial, antifungal, anthelmintic, and anticancer activities, as well as plasminogen activator inhibitor-1 inhibitory and anti-hyperglycemic effects [1]–[2]. DKPs also exhibit various other activities related to the nervous system such as antagonizing calcium channels and opioid, serotonin 1A, and oxytocin receptors and modulating the gamma-aminobutyric acid receptor [1]–[2]. One DKP, cyclo(L-His- L-Pro), is mainly produced from the precursor thyrotropin releasing hormone (TRH) protein, (L-(pyro)Glu-L-His-L-Pro-NH_2_) in mammals [2] and retains the neuroprotective activity across multiple animal trauma models [12]. Derivatives of cyclo(L-His- L-Pro) have been studied extensively to develop therapeutic agents for neuronal degeneration [13].

Chicken essence beverages are consumed by Southeast Asians as traditional remedies for several ailments, as a nutritional supplement for the elderly, and by students to reduce test-taking anxiety. Evidence indicates that chicken essence reduces anxiety in human subjects [14]. Patients diagnosed with anxiety disorders, according to the revised Diagnostic and Statistical Manual, Fourth Edition revised criteria [15], experienced significant improvements in their anxiety level, systolic blood pressure, and pulse rate when given chicken essence in combination with psychotherapy.

We have found that a chicken essence beverage inhibited serotonin transporter (SERT) and suppressed serotonin (5-HT) uptake from rat brain synaptosomes. Hence, we purified and identified the active ingredient from the chicken essence beverage by monitoring SERT inhibitory activity. We successfully purified the SERT inhibitory activity and identified cyclo(L-Phe-L-Phe) as an active ingredient. Using commercial synthetic cyclo(L-Phe-L-Phe), the ingredient was shown to be a naturally-occurring dual inhibitor that inhibited both SERT and acetylcholinesterase (AChE) *in vitro*. We further confirmed that oral administration of cyclo(L-Phe-L-Phe) increased cerebral monoamine levels and significantly improved depressive behavior in mice in a depressed state induced by the open-space swimming procedure and ameliorated scopolamine-induced learning and memory impairment in rats and mice. These data suggested that cyclo(L-Phe-L-Phe) is a dual inhibitor of the SERT and AChE that improves both depression and dementia.

## Results

### Purification and Identification of the Active Ingredient From a Chicken Essence Beverage

**Figure 1:**
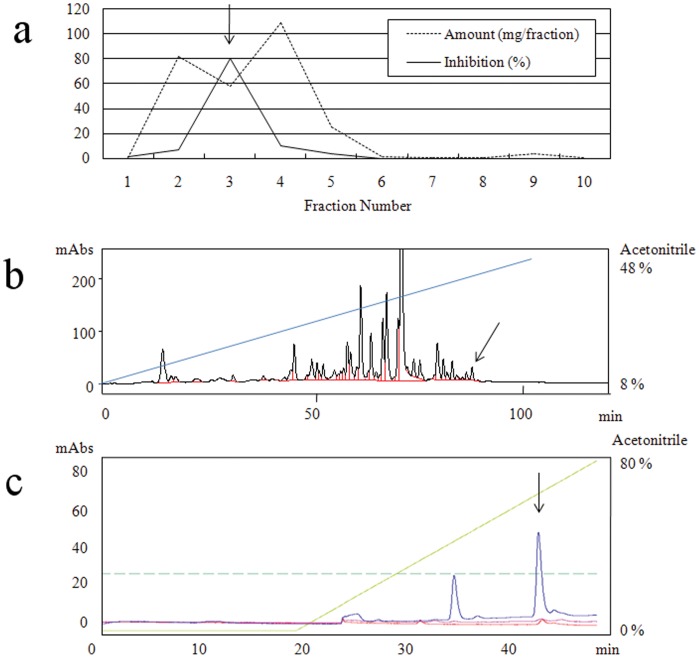
Purification of a serotonin transporter (SERT) inhibitor from a chicken essence beverage. (A) Sephadex LH-20 column chromatography of the diethyl ether extract. Every 80 ml of elution was collected from a Sephadex LH-20 column. Dried weight (dotted line) and SERT inhibitory activity (solid line) are shown. (B) Develosil ODS-HG-5 column chromatography of the fraction 3 from (A). The column was eluted with a linear gradient of 8–48% acetonitrile at a flow rate of 6 ml/min (blue line). The black line represents the relative absorbance at 215 nm. The red line represents peak fractionation. The active peak shown by the arrow was evaporated and dissolved in distilled water. (C) SOURCE 15 RPC column chromatography of the active fraction in (D). The column was eluted with a linear gradient of 0–80% acetonitrile at a flow rate 0.5 ml/min (green line). The blue, pink, and red lines represent the relative absorbance at 215, 240, and 280 nm, respectively. Dotted blue-green line represents 25 milliabsorbance (mAbs). The active peak is shown by the arrow.

Chicken essence powder was dissolved in 1 M acetic acid, extracted with diethyl ether, and purified as described in Materials and Methods. Briefly, SERT inhibitory activity was eluted at 160–240 ml from a Sephadex LH-20 column (Fig. 1A, fraction number 3) and then applied to a Develosil ODS-HG-5 column. The elution profile is shown in Fig. 1B. The activity peak shown by the arrow was finally applied to a SOURCE 15 RPC column. The peak SERT inhibitory activity fraction shown by the arrow (Fig.1C) was analyzed by mass spectrometry and nuclear magnetic resonance and yielded pure cyclo(L-Phe-L-Phe) as an active ingredient (data not shown). We confirmed that commercial synthetic cyclo(L-Phe-L-Phe) (Bachem AG, Bubendorf, Switzerland) inhibited SERT with an IC_50_ of 8.1 µM *in vitro*. In contrast, the norepinephrine transporter (NET) was not inhibited, indicating that cyclo(L-Phe-L-Phe) is a selective serotonin reuptake inhibitor (SSRI) (data not shown). We used synthetic cyclo(L-Phe-L-Phe) as the active compound in subsequent experiments.

### Measurement of the Apparent Permeability Coefficient (Papp) using a Blood–brain Barrier (BBB) *in vitro* Model

**Figure 2:**
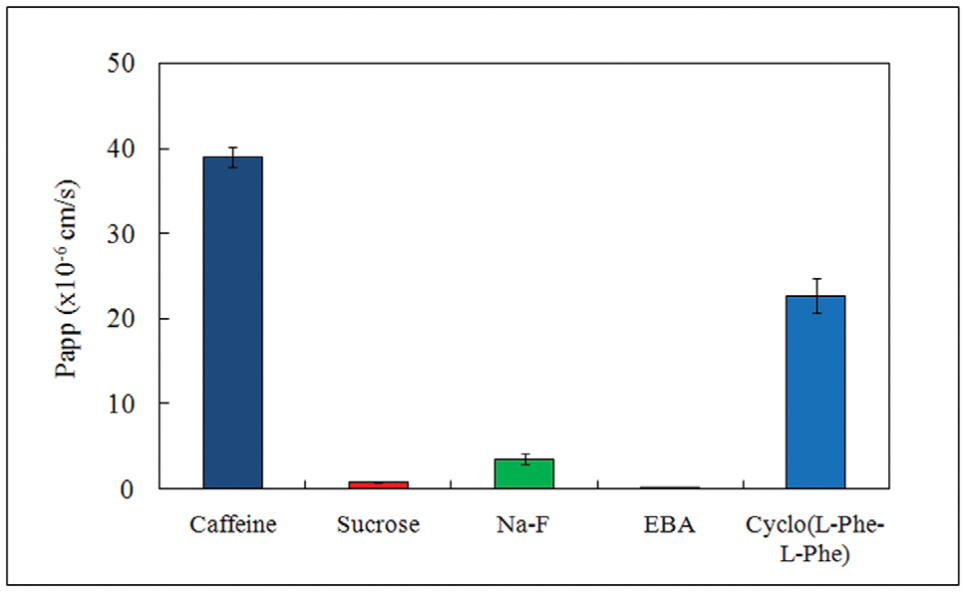
Apparent permeability coefficient (Papp) of cyclo(L-Phe-L-Phe) measured using a blood–brain barrier (BBB) *in vitro* model. Papps of caffeine, sucrose, Na-F, Evans blue albumin (EBA), and cyclo(L-Phe-L-Phe) were measured and calculated.

Cyclo(L-Phe-L-Phe) was apparently lipophilic based on its molecular structure and extractability with diethyl ether, and, as expected, was able to cross the BBB. It has been reported that *N*-methyl derivatives of DKPs easily crossed the BBB and can be used as BBB–shuttles [16] which could facilitate transport of therapeutic substances across the BBB. We measured the Papp in a BBB *in vitro* model using primary cultures of three rat cerebral microvessels cell types (endothelial cells, pericytes, and astrocytes) [17]. As shown in Fig. 2, Evans blue albumin (EBA) did not cross the BBB, whereas sucrose was only able to slightly cross and some Na-F crossed the BBB. In contrast, caffeine and cyclo(L-Phe-L-Phe) crossed the BBB readily. The Papp of cyclo(L-Phe-L-Phe) was relatively high and reached 58.3% of the levels of caffeine, which has a very high permeability to the BBB and is a central nervous system stimulant. These results warranted further investigations to study the effects of cyclo(L-Phe-L-Phe) on brain functions *in vivo*.

### Effect of cyclo(L-Phe-L-Phe) on Cerebral Monoamine Levels in the Medial Prefrontal Cortex (mPFC) and Acetylcholine (ACh) Levels in the Ventral Hippocampus (VHIPP) of Rats

**Figure 3:**
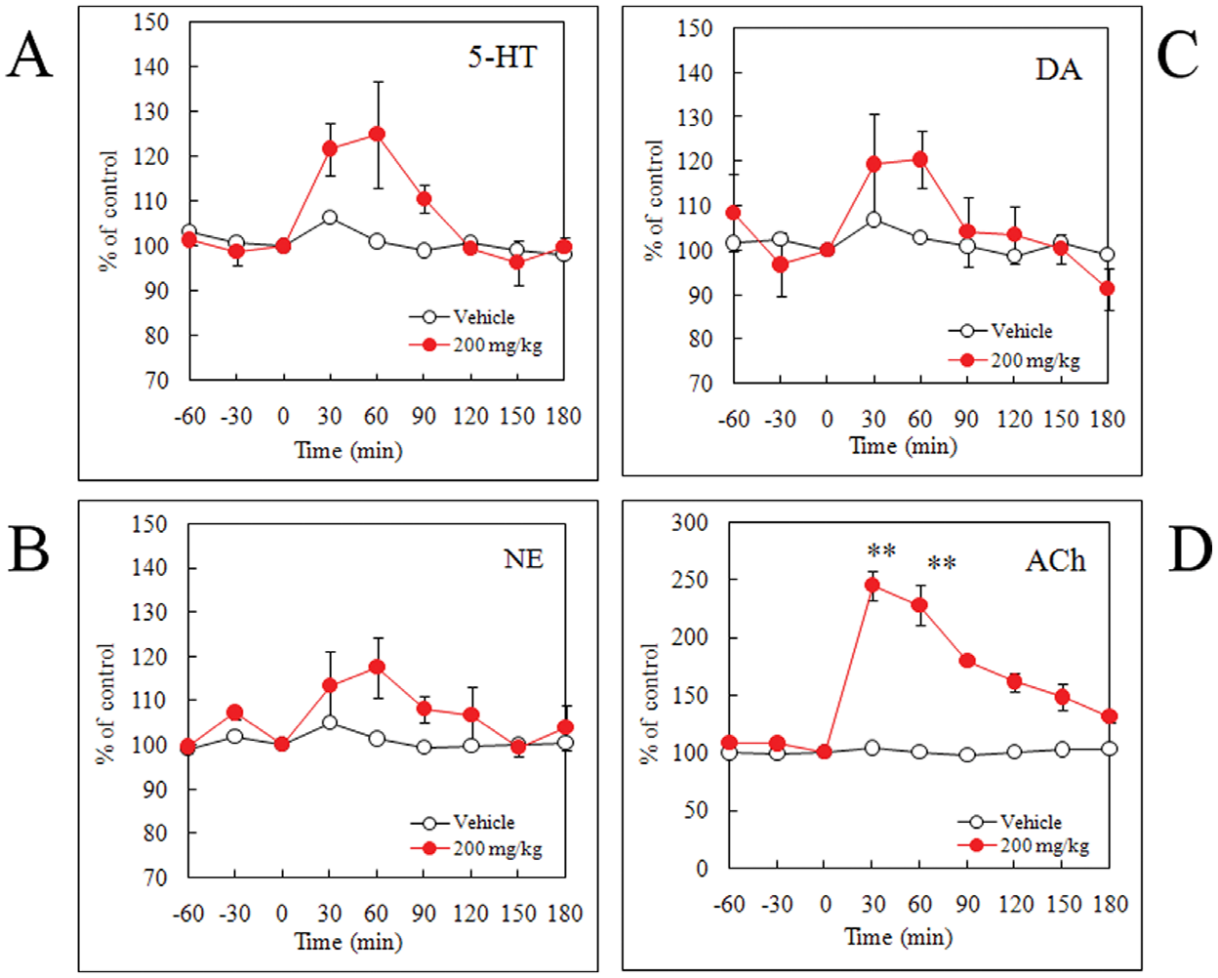
Effects of single administration of cyclo(L-Phe-L-Phe) on extracellular levels of serotonin (5-HT) (A), norepinephrine (NE) (B), and dopamine (DA) (C) in the medial prefrontal cortex (mPFC) and extracellular levels of acetylcholine (ACh) (D) in the ventral hippocampus (VHIPP) of freely moving rats measured using brain microdialysis. Male Sprague–Dawley rats were orally administered 200 mg/kg cyclo(L-Phe-L-Phe). Values within the treated groups were compared to those of the vehicle group at respective time points (two-way analysis of variance followed by Bonferroni’s post-test; **P<0.01. All values are mean ± standard error, n = 6 rats).

We examined the effects of cyclo(L-Phe-L-Phe) on cerebral monoamine levels in the mPFC and ACh levels in the VHIPP of freely moving rats using brain microdialysis. A single oral administration of cyclo(L-Phe-L-Phe) at 200 mg/kg slightly, but not significantly, increased extracellular levels of 5-HT, norepinephrine (NE) and dopamine (DA) in the mPFC (Fig. 3 A, B, C). However, the same cyclo(L-Phe-L-Phe) dose significantly increased extracellular levels of ACh in the VHIPP at 30 and 60 min after administration (*P*<0.01) (Fig. 3D).

**Figure 4:**
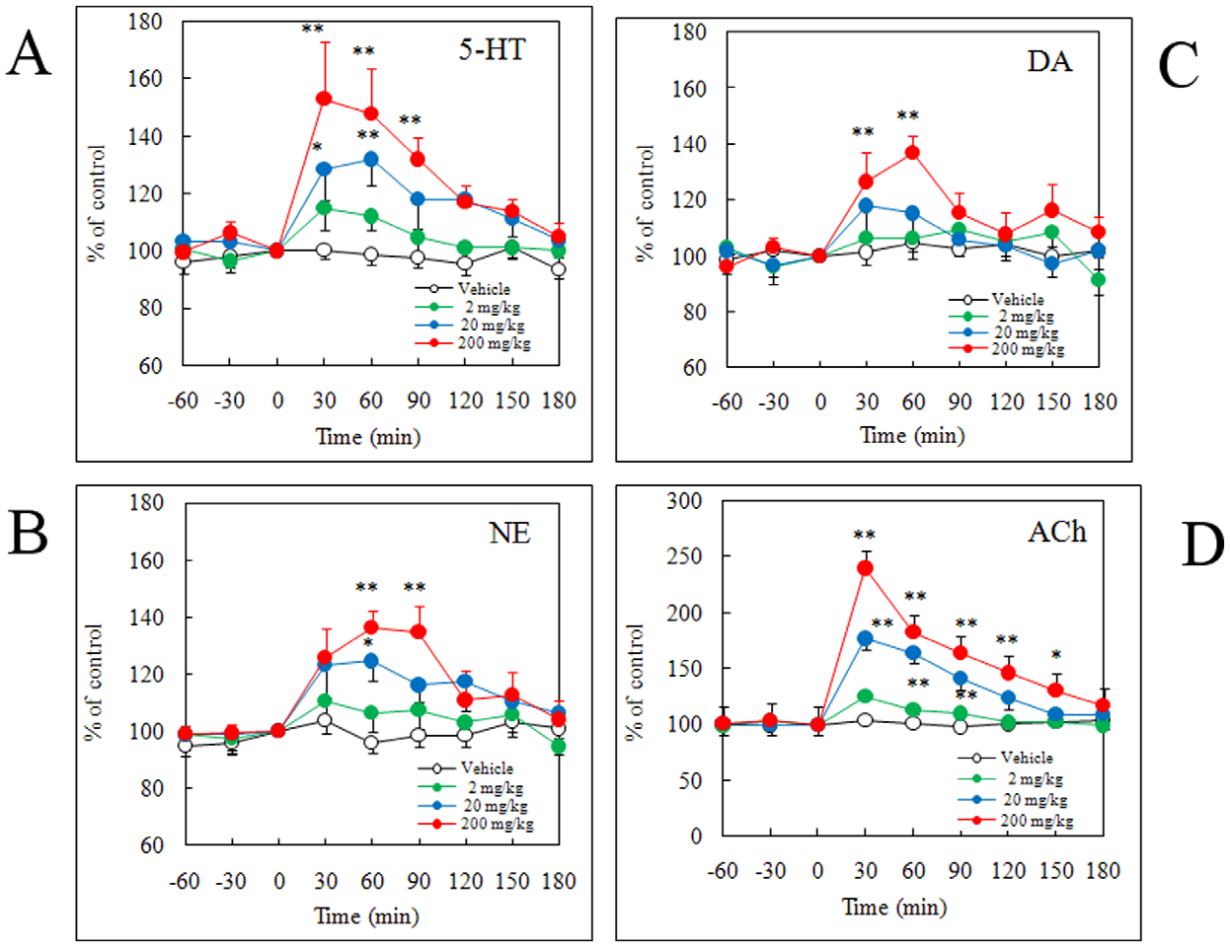
Effects of a 14-day administration of cyclo(L-Phe-L-Phe) on extracellular levels of serotonin (5-HT) (A), norepinephrine (NE) (B), and dopamine (DA) (C) in the medial prefrontal cortex (mPFC) and extracellular levels of acetylcholine (ACh) (D) in the ventral hippocampus (VHIPP) of freely moving rats measured using brain microdialysis. Cyclo(L-Phe-L-Phe) was orally administered to male Sprague–Dawley rats at doses of 2, 20, and 200 mg/kg after sub-chronic treatment for 13 consecutive days once daily at the same doses. Values of the treated groups were compared to that of the vehicle group at respective time points (two-way analysis of variance followed by Bonferroni’s post-test; *P<0.05, **P<0.01. All values are mean ± standard error, n = 6 rats).

When cyclo(L-Phe-L-Phe) was administered once daily for 14 consecutive days, significant increases in extracellular levels of 5-HT, NE, and DA were observed in the mPFC (Fig. 4A, B, C). In particular, administration of cyclo(L-Phe-L-Phe) caused a dose-dependent increase in 5-HT levels within the first 120 min after the last administration. The greatest increase in 5-HT level reached 152.9% of that of the control at 30 min after administering the highest dose (200 mg/kg) (*P*<0.001), and the levels remained significantly high during the following hour, 147.9% of that of the control at 60 min (*P*<0.001) and 131.9% at 90 min (*P*<0.01) (Fig. 4A). The typical SSRIs, fluoxetine, paroxetine, and sertraline, also significantly increased the release of 5-HT, NE, and DA in the mPFC [18], suggesting that cyclo(L-Phe-L-Phe) influenced the frontocortical monoaminergic pathways by similar reciprocal cerebral interactions. Repeated administration of 20 and 200 mg/kg cyclo(L-Phe-L-Phe) caused a significant increase in extracellular ACh concentrations at 30 (*P*<0.001) and 60 min (*P*<0.05) (Fig. 4D). Peak levels were observed at 30 min after administration, and ACh levels increased 177% and 239%, after administration of 20 and 200 mg/kg, respectively.

### Measurement of AChE Inhibitory Activity

We conducted *in vitro* AChE assays using the AChE highly selective substrate 1,1-dimethyl–4-acetylthiomethylpiperidinium iodide to clarify whether cyclo(L-Phe-L-Phe) affects synthesis, degradation, or release of ACh. Cyclo(L-Phe-L-Phe) inhibited AChE activity with an IC_50_ of approximately 3.4 µM, indicating that ACh accumulation was caused by inhibiting its degradation. These results clearly show that cyclo(L-Phe-L-Phe) is a dual inhibitor of the SERT and AChE.

### Water Maze Test Using Depressed Mice Previously Subjected to Repeated Open-space (OS) Swimming

**Figure 5:**
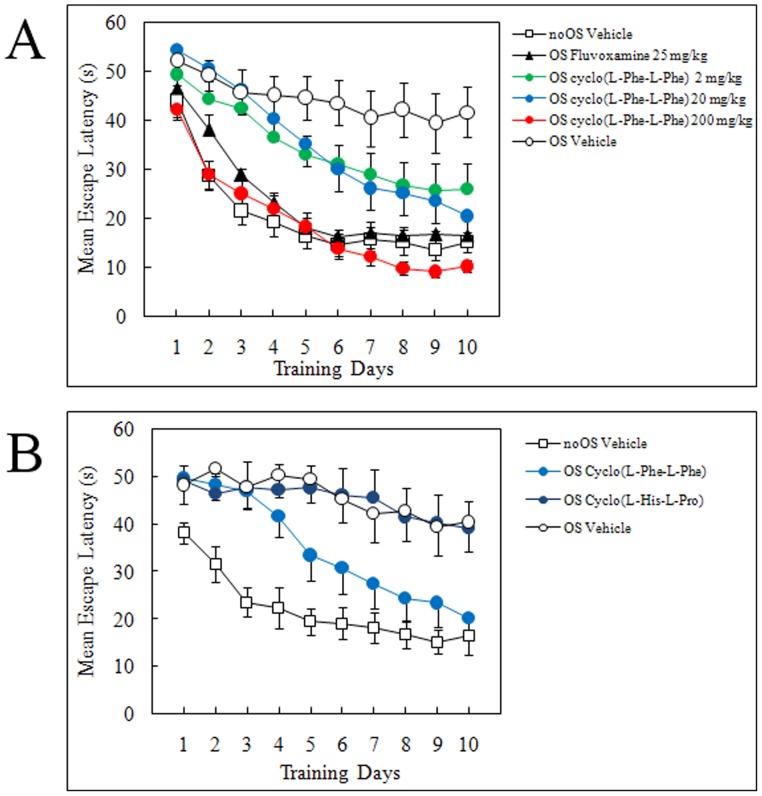
Effects of cyclo(L-Phe-L-Phe) and cyclo(L-His-L-Pro) on escape latency in the water maze test using depressed mice previously subjected to repeated open swimming (OS). (A) Cyclo(L-Phe-L-Phe) was orally administered at doses of 2, 20 and 200 mg/kg. Experimentally naïve male C57BL/6N mice were used. Vehicle control mice treated with/without training trials were represented as the OS vehicle and nonOS vehicle, respectively. The selective serotonin reuptake inhibitor (SSRI) fluvoxamine maleate (25 mg/kg, i.p.) was used as the positive control. (B) Cyclo(L-Phe-L-Phe) and cyclo(L-His-L-Pro) were orally administered at dose of 20 mg/kg. All values are mean ± standard error (n = 10–13 mice).

Next, we examined the effects of cyclo(L-Phe-L-Phe) on animal behavior to shed light on its mental health effects. We conducted a forced swimming test in rats and mice to investigate the antidepressant effect of cyclo(L-Phe-L-Phe). The forced swimming test is commonly used to examine antidepressant efficacy; however, SSRIs demonstrate limited effects in this test [19]–[20]. In fact, the tricyclic antidepressant imipramine shortened immobility time in the forced swimming test, whereas 25 mg/kg fluvoxamine maleate administered intraperitoneally or 2 mg/kg of cyclo(L-Phe-L-Phe) administered orally had no effect on immobility time (data not shown). Second, we conducted the water maze test using depressed mice previously subjected to repeated OS swimming, which induces a depression-like state in rats and mice [21]–[23]. Antidepressants such as SSRIs were effective in this behavioral test. Actually, the escape latency in the water maze test of mice pretreated with OS swimming and repeatedly given fluvoxamine orally at a dose of 25 mg/kg decreased to the level of control mice (Fig. 5A). Similarly, repeated administration of cyclo(L-Phe-L-Phe) ameliorated the depressed state, as shown by the decreased escape latencies (Fig. 5A). The highest dose tested, *i.e.* 200 mg/kg, cyclo(L-Phe-L-Phe), was as effective as 25 mg/kg fluvoxamine, whereas less but significant effects were observed at doses of 2 and 20 mg/kg (Fig. 5A). The effects of group (*F*_5, 67_ = 10.85), training day (*F*_9, 603_ = 71.88), and their interaction (*F*_45, 603_ = 2.09) were all significant (*P*s <0.0001). Furthermore, *post-hoc* analyses revealed significant differences between the OS vehicle and the other groups (*P*s <0.05). Collectively, our results demonstrate that the cognitive performance of depressed mice was significantly impaired, and that cyclo(L-Phe-L-Phe) ameliorated the impaired performance.

Figure 5B shows the results of the water maze test conducted using another DKP, cyclo(L-His-L-Pro). We selected the DKP because we found that among DKPs tested, this alone inhibited SERT albeit at much higher concentrations (IC_50_, 2.5 mM). Oral administration of 20 mg/kg of cyclo(L-His-L-Pro) resulted in no activity, whereas the same dose of cyclo(L-Phe-L-Phe) significantly ameliorated impaired performance (Fig. 5A, 5B).

### Radial Maze Test in Rats and Step-through Passive Avoidance Test in Mice

We conducted the radial maze test in rats [24]–[25] and step-through passive avoidance test in scopolamine-impaired mice [26] to investigate the anti-dementia effects of cyclo(L-Phe-L-Phe). Scopolamine interferes with memory and cognitive function in humans and experimental animals by blocking muscarinic ACh receptors [27]. Each arm in the radial maze test contains a food reward, and the rat is required to visit each arm once within a trial to locate all eight food rewards. Therefore, the task requires the animal to keep track of the already visited arms by making use of visuospatial cues located in the environment [28]. A repeated visit to the same arm was counted as a working memory error during a test session.

**Figure 6:**
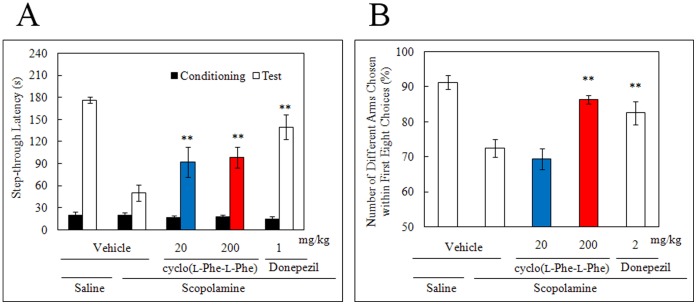
Effects of cyclo(L-Phe-L-Phe) on the number of different arms chosen within the first eight trials in the radiaL-arm maze test (A) and the step-through latencies in the passive avoidance test (B) with scopolamine-induced learning impairment. Male Sprague–Dawley rats (6-weeks-old) and male C57BL/6N mice were used in the radiaL-arm maze and the passive avoidance tests, respectively. Two doses (20 and 200 mg/kg) of cyclo(L-Phe-L-Phe) suspended in 0.5% CMC-Na were administered orally 10 min before scopolamine treatment. Cyclo(L-Phe-L-Phe) was orally administered at doses of 20 or 200 mg/kg 30 min after each scopolamine administration. Donepezil (1 mg/kg in the radial maze test or 2 mg/kg in the passive avoidance test) suspended in 0.5% CMC-Na was intraperitoneally administered as the positive control, and 0.5% CMC-Na was used as the vehicle control. All values are shown as mean ± standard error (Bonferroni’s post-test; **P<0.01, n = 10 rats in the radial maze test and n = 10–16 mice in the passive avoidance test).

We found that 2 mg/kg of the positive control donepezil and 200 mg/kg of orally administered cyclo(L-Phe-L-Phe) significantly improved the number of different arms chosen within the first eight choices during the radial maze test (Fig. 6A). In contrast, 20 mg/kg cyclo(L-Phe-L-Phe) had no improving effect (Fig. 6A), but the group effect was significant (*F*_4, 45_ = 13.47, *P*<0.0001). A significant difference was observed between groups of mice treated with cyclo(L-Phe-L-Phe) and those treated with scopolamine alone (*P*<0.001).

Next, we conducted the passive avoidance test (Fig. 6B). Electric shock during the conditioning trial prolonged the step-through latencies of the control mice during the retention test trial. However, scopolamine impaired acquisition of this passive avoidance behavior. Cyclo(L-Phe-L-Phe) at doses of 20 and 200 mg/kg resulted in significant increase in step-through latency along with the 1 mg/kg positive control donepezil (Fig.6A). The main effects of group (*F*_4, 63_ = 7.92), day (*F*_1, 63_ = 172.34), and their interaction (*F*_4, 63_ = 8.97) were significant (*P*s <0.0001). Bonferroni’s post-test conducted for groups on the retention test day revealed significant differences between groups of mice treated with cyclo(L-Phe-L-Phe) and those treated with scopolamine alone (*P*<0.01).

In summary, we demonstrated that acute administration of cyclo(L-Phe-L-Phe) (20 or 200 mg/kg) reversed the passive avoidance response during scopolamine-induced amnesia.

Taken together, our results clearly indicate that cyclo(L-Phe-L-Phe) has antidementia activity and acted as an antidepressant.

## Discussion

Our results show, for the first time, that the chicken essence beverage-derived DKP cyclo(L-Phe-L-Phe) is a dual inhibitor of the SERT and AChE. Because DKPs seem almost ubiquitous in fermented or processed foods and beverages and are present in many organisms, we tested whether other DKPs could inhibit SERT and AChE. We found that cyclo(L-His-L-Pro), a compound endogenous to animals that shares the C-terminal dipeptide sequence with TRH, inhibited SERT (IC_50_, 2.5 mM) but not AChE, and that cyclo(L-Pro-L-Pro), one of the bitter components of beer, inhibited AChE (IC_50_, 160 µM) but not SERT. However, cyclo(L-His-L-Pro) did not improve depression symptoms (Fig. 5B), possibly because of its much weaker affinity for SERT than that of cyclo(L-Phe-L-Phe). In contrast, oral administration of 200 mg/kg of cyclo(L-Pro-L-Pro) significantly increased extracellular levels of ACh in the VHIPP (data not shown). However, the same cyclo(L-Pro-L-Pro) dose failed to improve the number of arms chosen within the first eight choices during the radial maze test (data not shown), possibly because it had behavioral suppressing effects (our unpublished observation).

The affinities of cyclo(L-Phe-L-Phe) against SERT and AChE were fairly low compared with those of medical drugs such as the SERT inhibitors fluvoxamine and paroxetine and the AChE inhibitor donepezil. However, single oral administration at the dose of 200 mg/ml cyclo(L-Phe-L-Phe) showed a tendency to increase 5-HT, NE, and DA in the mPFC, although the difference was not significant (Fig. 3A, B, C). However, extracellular ACh in the VHIPP increased significantly (Fig. 3D). Furthermore, significant increases in extracellular levels of 5-HT, NE, DA, and Ach were observed dose-dependently when once per day administration was continued for 14 consecutive days (Fig. 4). Emerging evidence indicates that intact or naïve animals *vs.* trained animals show different results regarding the effectiveness of SSRIs [29]–[30]. Such complex responses to drugs might also underlie the difference between *in vitro* and *ex vivo* results.

Neverthless, cyclo(L-Phe-L-Phe) was effective in several animal behavioral tests that a reflect states of depression or dementia (Figs. 5 and 6). These results clearly suggest that despite its comparatively low inhibitory activities against SERT and AchE *in vitro*, cyclo(L-Phe-L-Phe) attained high enough concentrations to show beneficial effects on brain functions if taken daily.

In view of human use, these moderate cyclo(L-Phe-L-Phe) effects may be important and desirable characteristics of food and beverage ingredients, which are taken almost daily. Cyclo(L-Phe-L-Phe) is of special interest among DKPs in foods and beverages because it is found not only in chicken essence [11] but also in some commercial chicken soups such as bouillon cubes and powders. Besides its known biological activities (antifungal [31] and anthelmintic activities [32]), we have now shown that cyclo(L-Phe-L-Phe) is a dual inhibitor of the SERT and ACh that has both antidepression and antiamnesic effects in animal models. Animal studies have demonstrated that post-training SSRI administration improves memory more than that of pre-training administration [33]. Increasing evidence indicates a close relationship between depression and cognitive deficits in human patients [34]–[35]. Depression is not only an emotional disorder, but also has negative impacts on learning and memory abilities.

Dementia has become a serious problem in many countries in recent years because of the rapid aging of society [36]. Alzheimer’s disease (AD) is a typical dementia caused by neurodegeneration. Approximately 40–50% of patients with AD suffer from depressive state, and 10–20% are complicated by depression [37]. Among patients with vascular dementia, 60% suffer from a depressive state, and 27% are complicated by depression [38]. Apparently, depression is a risk factor for various types of dementia, including AD [39]. Dual SERT and AChE inhibitors are being developed to treat both depression and dementia with a single agent [40].

Our present findings indicate that cyclo(L-Phe-L-Phe) may be a good therapeutic candidate for treating depression and dementia. As mentioned above, another DKP, cyclo(L-His- L-Pro), endogenous to animals has been derivatized and studied extensively as a therapeutic agent of neuronal degeneration. However, a more important implication may be that daily consumption of the chicken essence, which contains cyclo(L-Phe-L-Phe), might help people stay mentally healthy in an increasingly aging societies.

## Materials and Methods

The microdaialysis experiments were conducted by Pronexus Analytical AB (Stockholm, Sweden). These animal experiments follow the directives of the “Principles of Laboratory Animal Care” (NIH publication No. 85-23) and the Council of the European Communities (86/809/EEC) and were approved by the Swedish national ethical committee (Stockholms Norra djurforsoksetiskanamnd), according to established protocols and Pronexus Standard Operation Procedures (approval ID: N191/10). The water maze learning task after the OS swimming and passive avoidance test were conducted by Kouiken Co. Ltd. (Hyogo, Japan). The animal experiments were conducted according to “Guide for Care and Use of Laboratory Animals” published by the National Institutes of Health (NIH) and approved by the “Ethical committee of Behavioral and Medical Science Research Consortium”. The radial maze experiment was conducted by Charles River Laboratories Japan, Inc. (Osaka, Japan) (approval ID: 39/42/55/57). The animal experiment was conducted according to “Guide for Care and Use of Laboratory Animals” published by the NIH and approved by the “Institutional Animal Care and Use Committees of Charles River Japan” (approval ID: 483).

### Purification of a Dual Inhibitor from a Chicken Essence Beverage

Sephadex LH-20 media and a SOURCE 15 RPC column (4.6×100 mm ) were purchased from GE Healthcare Japan (Tokyo, Japan). A Develosil ODS-HG-5 column (4.6×250 mm) was purchased from Nomura Chemicals Co. Ltd. (Aichi, Japan). Chicken essence powder (135 g) was dissolved in 1 M acetic acid at 200 mg/ml and mixed with an equal volume of diethyl ether. The resulting diethyl ether layer was collected, dried, dissolved in 80% acetonitrile/distilled water, and loaded onto a Sephadex LH-20 column (6×18 cm) equilibrated with the same buffer. Isocratic elution was carried out, and eluted fractions were collected for every 80 ml volume. The active fraction (fraction 3 of Fig. 1a) was further dried, dissolved in 8% acetonitrile/distilled water (DW), and loaded onto a Develosil ODS-HG-5 column equilibrated with the same buffer. The weights of the dried diethyl ether extract and fraction 3 of Fig. 1a were 467 mg and 81.6 mg, respectively. The column was eluted with a linear gradient of 8–48% acetonitrile at a flow rate of 6 ml/min. The active peak shown by the arrow was evaporated, dissolved in DW, and loaded onto a SOURCE 15 RPC column equilibrated with DW. The column was eluted with a linear gradient of 0–80% acetonitrile at a flow rate 0.5 ml/min. The active peak fraction shown by the arrow was evaporated, dissolved in DW, and further characterized.

### SERT, NET, and AChE Inhibition Assays

SERT and NET inhibition assays were conducted as described previously [41]–[42]. The AChE inhibition assay was conducted using a highly selective substrate AChE MATP+ (1,1-dimethyl–4-acetylthiomethylpiperidinium iodide (Dojindo Laboratories, Kumamoto, Japan), the indicator DNTB (2,4-Dinitro-1-thiocyanobenzene), and AChE (amphiphilic form from human erythrocytes; Sigma, St. Louis, MO, USA). Sample (30 µl) was pre-incubated with 30 µl of 0.5 U/ml AChE in a 96–well plate for 15 min at room temperature (RT), and a 20 µl aliquot of the pre-incubation mixture was mixed with 80 µl of the substrate mixture (1.875 mM MATP+ and 1.25 mM DNTB) in a 96–well plate. The assay mixture was left to stand for 2 h at RT before absorbance was measured at a wavelength of 412 nm using a Shimadzu UV 160A spectrophotometer (Shimadzu Corp., Kyoto, Japan). Tris-HCl buffer was used as the blank.

### Measurement of Papp Using a BBB *in vitro* Model

A BBB *in vitro* model (BBB kit) prepared with primary rat brain capillary endothelial cells, brain pericytes, and astrocytes was purchased from PharmaCo-Cell Company Ltd. (Nagasaki, Japan) and used as described previously [17]. A 12–well dish from the kit was pre-incubated at 37°C to thaw cells, according to the manufacturer’s instructions. The sample (caffeine, sucrose, Na-F, EBA, or cyclo(L-Phe-L-Phe)) in Dulbecco’s Modified Eagle’s Medium (Life Technologies Japan, Tokyo, Japan) was added to the blood-side of a well at a final concentration of 1 µM. After a 20, 40, or 60 min incubation, aliquots of the solutions were collected from both luminal and abluminal sides. Sample concentrations were measured, and Papp was calculated according to manufacture’s protocol.

### Surgery and Microdialysis

The microdialysis experiments were conducted in awake, freely moving rats following a protocol described previously [43]–[45]. Briefly, 9–11 week-old male Sprague–Dawley rats were anaesthetized with isoflurane using a Univentor 400 anesthesia unit (AgnThos, Lidingö, Sweden) and placed in a stereotaxic frame (David Kopf Instruments, Tujunga, CA, USA) in a flat skull position with the incisor bar set to −3.2 mm. The body temperature of the animals was controlled and maintained at 37°C using a CMA/150 temperature controller (CMA/Microdialysis, Stockholm, Sweden) and a rectal thermometer. One hole for the guide cannula and three holes for the anchor screws were drilled using a fine trephine drill. The guide cannula for the microdialysis probe (Eicom Corp., Kyoto, Japan) was implanted into the mPFC or the VHIPP according to the atlas of Paxinos and Watson [46]. The entire assembly was secured with dental cement (Dentalon Plus, Heraeus, Germany), and the rats were allowed to recover for 5 days. On the day of the experiment, the microdialysis probe (0.22 mm o.d., 4 mm membrane length with 50,000 Da cut-off, Eicom CX-I, Eicom Corp.) was inserted into the guide cannula of the awake rat.

The test compound was orally administered through a gavage once daily for 13 days during the repeated administration experiment. The body weights and the general status of the animals were monitored on a regular basis during this periods. On the day of the experiment, the microdialysis probe was inserted into the guide cannula of an awake rat. The inlet and outlet tubing were connected to a 1 ml syringe mounted to a CMA/102 microinjection pump (CMA/Microdialysis) and an Eicom fraction collector. The probe was perfused with artificial cerebrospinal fluid solution (148 mM NaCl, 4 mM KCl, 0.8 mM MgCl_2_, 1.4 mM CaCl_2_, 1.2 mM Na_2_HPO_4_, 0.3 mM NaH_2_PO_4_, pH 7.2) at a flow rate of 1 µl/min. Samples were collected every 30 min after 2 h stabilization period, using an Eicom fraction collector. The first three samples were taken to determine basal extracellular levels of monoamines and ACh. Thereafter, the test compound was orally administered by gavage, and fractions were collected for an additional 180 min. After termination of the experiment, the rats were sacrificed, and the brains were removed for histological verification of microdialysis probe placement.

### High-performance Liquid Chromatography (HPLC)

5-HT, NA, and DA were determined by narrow-bore column liquid chromatography using electrochemical detection. The chromatographic conditions were optimized to allow simultaneous determination of all three monoamines in the same sample. ACh was determined by HPLC linked to a post-column immobilized enzyme reactor followed by electrochemical detection, as described previously [43]–[44]. The HPLC system (HTEC-500, Eicom) included a pulse-free microflow pump, a degasser, and an amperometric detector equipped with a graphite electrode operating at +0.45 V vs. an Ag/AgCl reference electrode. Samples were injected using a CMA/200 Refrigerated Microsampler (CMA/Microdialysis), and chromatograms were recorded and integrated using a computerized data acquisition system (DataApex, Prague, Czech Republic). 5-HT, NA, and DA were separated on a 200×2.0 I.D. mm column (CAX, Eicom). The mobile phase consisted of 0.1 M phosphate buffer at pH 6.0, 30 mM potassium chloride, and 28% methanol. The detection limits (signaL-to-noise ratio = 3) for 5-HT, NA, and DA were 0.5, 0.55, and 0.45 fmol respectively, and 15 µl was injected onto the column. ACh and choline were separated on a 150×2.0 I.D. mm narrow-bore column, packed with 4 µm size (Eicom) C18 polymer gel. An AC-ENZYMPAK II enzyme reactor (Eicom) was used. The mobile phase was 50 mM potassium hydrogen carbonate solution containing 3.7 mM sodium 1-octanesulfonate and 0.13 mM EDTA-2Na. The detection limit (signaL-to-noise ratio = 3) for ACh was about 3 fmol, and 15 µl was injected onto the column.

### Water Maze Learning Task After OS Swim [21]–[23]

An OS swimming procedure was used to induce a depression-like state in mice. Experimentally naïve male C57BL/6N mice were placed individually in a circular pool (diameter, 95 cm; height, 35 cm) that was filled to a 21 cm depth with water maintained at a temperature of 24±1°C. The pool was enclosed by white featureless walls (height, 120 cm), and almost all visual cues were removed. Mice were allowed to swim (or not swim) freely for 5 min daily for 5 consecutive days.

To assess the effects of cyclo(L-Phe-L-Phe) on the depressive behavior induced by OS swimming, the Morris water maze learning task [47] was performed using a circular pool (diameter, 95 cm; height, 35 cm) placed in a soundproof testing room with various extra-maze cues. The pool was filled with water to a 22 cm depth and made opaque by adding titanium oxide. The temperature of the water was maintained at 24±1°C. The water surface of the pool was divided into north, south, east, and west quadrants. A white round platform (diameter, 11.5 cm) was situated at the center of the north quadrant and submerged 0.5 cm below the water surface.

Each mouse was subjected to five training trials per day for 10 consecutive days. Cyclo(L-Phe-L-Phe) (2 or 20 mg/kg, p.o.) or vehicle was administered in a volume of 10 ml/kg 30 minutes prior to the start of daily training sessions. The mouse was released into the water in the center of the pool near the edge in the south, east, or west quadrant, with its head facing the outer edge of the pool. A training trial terminated when the mouse reached the platform and remained on it for 10 s. If the platform was not found within 60 s, the mouse was guided to the platform by the experimenter and kept there for 10 s. The order of the release points was varied on a daily basis, and pseudorandom sequences were used for each mouse. The inter-trial interval was 30 s. The escape latency in each trial was measured up to a maximum of 60 s. The SSRI fluvoxamine (25 mg/kg, i.p.) was used as the positive control.

### RadiaL-arm Maze

Spatial learning and memory were assessed by choice accuracy in an eight-arm radial maze, as described previously [48]–[49]. Briefly, 6 week-old male Sprague–Dawley rats were used. Rats had *ad libitum* access to water with daily feeding after testing to maintain body weight at a lean healthy weight with a target of approximately 75–80% of free feeding level. The black-painted wood maze was situated at an elevation of 30 cm. The central platform had a diameter of 50 cm, and eight arms (10×60 cm) projected radially outward. Training continued until rats entered into seven baited arms. The session lasted for up to 300 s or until rats entered all eight baited arms. Scopolamine amnesia was induced by injecting 0.5 mg/kg scopolamine intraperitoneally 30 min before each trial. Vehicle control mice received saline in the same way. Arms were baited only once, and repeated entry into a baited arm was counted as a working memory error. Entrance into an unbaited arm was recorded as a reference memory error. The angles between two successive arm choices were distributed equally from 45° to 180°. We confirmed that all groups of rats were not using an “adjacent arm” search strategy.

### Passive Avoidance Test [26]

Experimentally naïve male C57BL/6N mice were used. Each mouse was trained in a step-through type passive avoidance apparatus consisting of two compartments (10×10×20 cm each), one light and one dark with a grid floor. A guillotine door separated these two compartments. In the acquisition trial, the mouse was put in the light compartment, and the guillotine door was opened 5 s later. When the mouse spontaneously moved into the dark compartment, the guillotine door was closed and an electric shock (160V, 0.50 mA, 3 s) was delivered 10 s later through the grid floor. Latency to enter the dark compartment was recorded. A retention test trial was performed 24 h later. The mouse was placed back into the light compartment and the latency to re-enter the dark compartment was recorded up to a maximum of 180 s. No shock was delivered during the retention test trial. copolamine (0.5 mg/kg) was administered 30 min after the acquisition trial and 60 min before the retention test trial to induce amnesia.

### Statistical Analyses

Data are presented as mean ± standard error. Statistical analyses were performed using the *t*-test or analysis of variance with Bonferroni’s post-test. *P*–values <0.05 were considered significant.
